# Mucin 1, a signal transduction membrane-bound mucin, is present in human disc tissue and is downregulated in vitro by exposure to IL-1ß or TNF-α

**DOI:** 10.1186/s12891-017-1541-1

**Published:** 2017-05-08

**Authors:** Helen E. Gruber, Jane A. Ingram, Gretchen L. Hoelscher, Emilio Marrero, Edward N. Hanley

**Affiliations:** 1 Department of Orthopaedic Surgery, Carolinas Medical Center, PO Box 32861, Charlotte, NC USA; 2 Orthopaedic Research Biology, Carolinas Medical Center, Cannon Research Center, Room 304, PO Box 32861, Charlotte, NC 28232 USA

**Keywords:** Mucin 1, Intervertebral disc, Disc degeneration, Interleukin 1-ß, Tumor necrosis factor-α

## Abstract

**Background:**

Back pain and disc degeneration have a growing socioeconomic healthcare impact. Mucin 1 (MUC1) is a transmembrane glycoprotein whose extracellular and intracellular domains participate in cellular signaling. Little is currently known about the presence or role of MUC1 in human disc degeneration.

**Methods:**

In this IRB-approved research study, 29 human disc specimens were analyzed for MUC1 immunohistochemical localization and gene expression, and annulus fibrosus (annulus) cells were also isolated and cultured in 3D. Microarray analysis assessed expression levels of MUC1 in healthy and degenerated disc tissue and in cells exposed to proinflammatory cytokines (IL-1ß or TNF-α).

**Results:**

MUC1 was shown to be present in annulus cells at the protein level using immunochemistry, and its expression was significantly upregulated in annulus tissue from more degenerated grade V discs compared to healthier grade I-II discs (*p* = 0.02). A significant positive correlation was present between the percentage of MUC1-positive cells and disc grade (*p* = 0.009). MUC1 expression in annulus cells cultured in 3D was also analyzed following exposure to IL-1ß or TNF-α; exposure produced significant MUC1 downregulation (*p* = 0.0006).

**Conclusions:**

Here we present the first data for the constitutive presence of MUC1 in the human disc, and its altered expression during disc degeneration. MUC1 may have an important role in disc aging and degeneration by acting as a regulator in the hypoxic environment, helping disc cells to survive under hypoxic conditions by stabilization and by activation of HIF-1α as previously recognized in pancreatic cancer cells.

## Background

Mucin 1 (MUC1) is a transmembrane glycoprotein with a protein backbone of 20 amino acid residues repeated in tandem, a transmembrane sequence, and a 69 amino acid-long tail [4]. This conserved tail received post-translational modifications from growth factor receptor tyrosine kinases (RTKs), and can participate in interactions with factors which can directly influence transcriptional regulatory ability [3]. In non-polarized cells, MUC1 can make contact with the RTKs via its tail, and can then signal in response to growth and differentiation factors. In the tumor microenvironment, MUC1 signaling has been found to reprogram transcription of connective tissue growth factor (CTGF, also called CCN2), which is a strong mediator of extracellular matrix (ECM) remodeling and angiogenesis [3]. Tran et al. have recently suggested that the specialized cross-talk between CTGF and hypoxia inducible factor-1 (HIF-1) may be important in the avascular adult degenerating disc [22]. Stabilized expression HIF-1α is now recognized as one of the “healthy nucleus [pulposus] phenotypic markers” [7, 17], and knockout of HIF-1α has been shown to accelerate disc degeneration in a mouse model [24]. Chaika et al. have shown that MUC1 can facilitate recruitment of HIF-1α in an hypoxia-dependent manner, suggesting that MUC1 can act as an important regulator in the hypoxic environment, helping cells to survive under hypoxic conditions by its stabilization and activation of HIF-1α [5].

Because of the potential importance of MUC1in disc biology and degeneration, in the present work we searched for expression of MUC1 in human disc tissue and its immunohistochemical localization; we also tested for its expression when annulus fibrosus (annulus) cells cultured in 3D were exposed to either interleukin 1-beta (IL-1ß) or tumor necrosis factor-alpha (TNF-α), two proinflammatory cytokines that are well-recognized in disc degeneration [14, 15]. These proinflammatory cytokines can be bound in the extracellular matrix surrounding cells within the degenerating disc, resulting in higher localized regional concentrations [16].

## Methods

### Clinical study population

Experimental study of human disc specimens was approved in a prospective manner by the authors’ Human Subjects Institutional Review Board at Carolinas Medical Center. Informed consent was waived by the ethical board because disc tissue used in the present study was removed as part of routine surgical practice. As we have reported previously, our scoring of disc degeneration utilized a modified Thompson scoring system which incorporated author ENH’s radiologic, MRI and surgical findings. The Thompson disc grading system scores disc degeneration across the span of disc degeneration, from healthy discs (Thompson grade I) to discs with the most advanced degeneration (grade V, the most severe stage of degeneration) [21]. As we have reported previously, specimens from patients were derived from surgical procedures performed on individuals with herniated discs and degenerative disc disease. These specimens were transported to the laboratory in sterile tissue culture medium. Control non-surgical donor disc specimens were obtained via the National Cancer Institute Cooperative Human Tissue Network (CHTN); these specimens were shipped overnight to the laboratory in sterile tissue culture medium and processed as described below. Procurement of these control non-surgical specimens was included in our approved Institutional Review Board protocol.

### Gene expression analyses of MUC1 in human disc tissue and in cultured human annulus cells

#### Human disc tissue

As we have previously described, human disc tissue (Table 1) was snap frozen in liquid nitrogen, pulverized (BioPulverizer, BioSpec Products, Inc., Bartlesville, OK, USA), and homogenized via the FastPrep-24 instrument (MP Biomedicals L.L.C., Santa Ana, CA, USA). Total RNA (100 μg) was then harvested, reverse transcribed, amplified, labeled, fragmented and hybridized to the Affymetrix human U133 X3P microarray chips. We utilized the GCOS Affymetrix GeneChip Operating System (version 1.2, Affymetrix, Santa Clara, CA 95051) to determine gene expression levels of MUC1.

**Table 1:** Demographic features for subjects whose disc tissue was evaluated with immunohistochemistry or whose tissue or cells were utilized for molecular analysis

Subject number	Age	Thompson score	Vertebral level	Specimen type^a^	Experimental use^b^
1	34	I	Lumbar	CHTN	Mol
2	30	II	L3-4	CHTN	Mol
3	30	II	L3-4	CHTN	Mol
4	54	II	L4-5	Surgical specimen	Mol
5	21	II	L5-S1	Surgical specimen	Mol
6	40	II	L4-5	Surgical specimen	Mol
7	41	V	L4-5	Surgical specimen	Mol
8	57	V	C6-7	Surgical specimen	Mol
9	72	V	L4-S1	Surgical specimen	Mol
10	1	I	Lumbar	CHTN	Imm
11	Newborn	I	Lumbar	CHTN	Imm
12	21	II	L5-S1	Surgical specimen	Imm
13	24	II	L5-S1	Surgical specimen	Imm
14	41	II	L5-S1	Surgical specimen	Imm
15	29	III	L5-S1	Surgical specimen	Imm
16	53	III	L5-S1	Surgical specimen	Imm
17	54	III	L4-5	Surgical specimen	Imm
18	58	III	L2-3	Surgical specimen	Imm
19	39	IV	L4-5	Surgical specimen	Imm
20	56	IV	L2-3	Surgical specimen	Imm
21	59	IV	L2-3	Surgical specimen	Imm
22	78	IV	L3-4	Surgical specimen	Imm
23	39	V	L5-S1	Surgical specimen	Imm
24	56	V	L5-S1	Surgical specimen	Imm
25	62	V	L5-S1	Surgical specimen	Imm
26	59	IV	L4-5	Surgical specimen	3D and Mol
27	59	IV	L4-5	Surgical specimen	3D and Mol
28	57	III	C4-5	Surgical specimen	3D and Mol
29	54	IV	L3-4	Surgical specimen	3D and Mol

^a^ *CHTN* Cooperative Human Tissue Network, normal donor specimen, *OR* surgical specimen, *L* lumbar, *C* cervical

^b^ *Imm* immunohistochemistry on disc tissue, *3D and Mol* cells cultured in 3D and 3D mRNA harvested for microarray analysis, *Mol* human annulus tissue mRNA harvested for microarray analysis

#### Cultured cells

Annulus cells were established in monolayer culture [9], and expanded for use in 3D in a collagen sponge as previously described [13]. Cells were cultured over14 days in 3D with media changes using either control conditions (minimal essential medium plus 20% FBS) or experimental test conditions with addition of IL-1beta (10^-2^ pM) or TNF-alpha (10^3^ pM) (Table 1). Doses used were previously determined in our laboratory [8, 10]. As we have previously reported, RNA was harvested from cells at experiment conclusion and gene expression studies carried out using microarray analysis. Total RNA (100 μg) was harvested, reverse transcribed, amplified, labeled, fragmented and hybridized to the Affymetrix human U133 X3P microarray chips. The GCOS Affymetrix GeneChip Operating System (version 1.2, Affymetrix, Santa Clara, CA 95051) was used for determining gene expression levels of MUC1 (gene identifier Hs.89603.6.A1_3p_a_at).

#### Statistical analysis of microarray data

Statistical analyses utilized GeneSifterTM web-based software to analyze microarray data. Employing the GC-RMA (Robust multi-array average) method, Affymetrix “.cel” files were uploaded to the GeneSifterTM web site, normalized, and corrected for false discovery rate (FDR). As we have previously reported, statistical significance was determined using Student’s t-test (2 tailed, unpaired) and significance was set at *p* < 0.05). Fold change was set at 2.0. Additional statistical analyses were performed using unpaired t-tests and Mann-Whitney tests (GraphPad Instat 3, GraphPad Software, Inc., San Diego, CA). If data were not distributed normally, analyses utilized nonparametric statistical methods.

### Immunolocalization of MUC1

Paraffin sections from human discs or from cells cultured in 3D were cut at 4 μm (Table 1), collected on PLUS slides (Cardinal Health, Dublin, OH) and dried at 60 °C. Sections were deparaffinized using xylene (Cardinal) and then rehydrated through graded alcohols (AAPER, Shelbyville, KY) to distilled water. 3% H_2_O_2_ (Sigma, St Louis, MO) was used to block endogenous peroxidase. Sections were incubated for one hour with anti-MUC-1 (epithelial membrane antigen MA5-13168, ThermoFisher, Rockford, IL) at a 1:200 dilution. The secondary reagent utilized was Vector Immpress Reagent, Anti-Mouse Ig (Vector Laboratories, Burlingame, CA) for 30 minutes followed by DAB (Dako) for 5 minutes. Slides were rinsed in water, counterstained with light green, dehydrated, cleared and mounted with resinous mounting media. Mouse IgG (Dako, Carpenteria, CA) was used as a negative control; human colon tissue was used as a positive control.

The number of cells positive for immunolocalization of MUC1 was determined and the percentage of positive cells analyzed for its relationship with the Thompson grade of that disc tissue. The mean number of cells scored/specimen was 209 ± 51 (13) (mean ± S.D. (n)) and the range 149 to 295. *Standard statistical methods* were performed using GraphPad Instat 3 (GraphPad Software, Inc., San Diego, CA). Spearman’s correlation coefficient was calculated to test for the association of Thompson grade with the % of cells positive for Muc1 immunolocalization, and r^2^ was used to determine goodness of fit.

## Results

Table 1 describes the source of disc tissue for specimens utilized for immunolocalization of MUC1, for cell culture experiments, and for tissue gene expression studies.

**Fig. 1:**
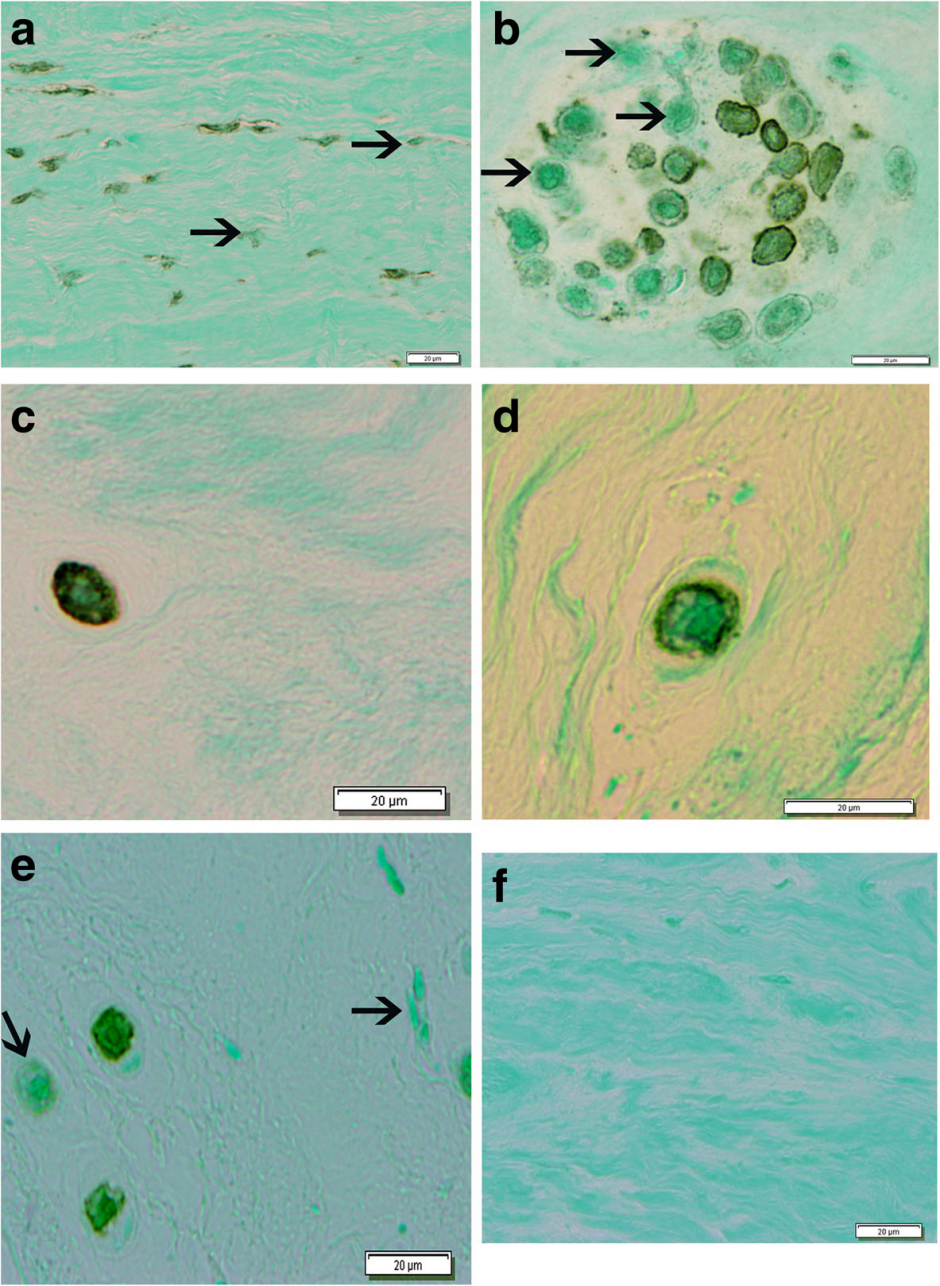
Representative images showing immunolocalization of MUC1. **a** Grade IV specimen shows the outer annulus, with many spindle-shaped cells. **b** Large cluster of cells in the inner annulus showing cells with positive or negative localization. **c** Cytoplasmic immunolocalization present in an inner annulus cell from a grade IV specimen. **d** Localization along the rim of the lacunar margin of a disc cell in the inner annulus. **e** Fewer immunopositive cells were present in the nucleus pulposus regions of the disc. **f** Negative control section adjacent to that shown in **a**. (Arrows mark cells which do not show localization. Bar = 20 μm)

**Fig. 2:**
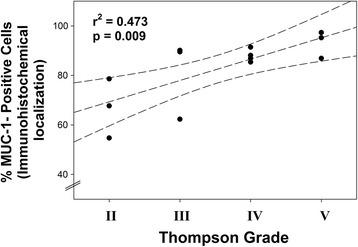
A significant, positive correlation was present between the proportion of cells exhibiting MUC1 immunolocalization and the disc Thompson grade (*r* ^2^ = 0.473; *p* = 0.009). (Dashed lines show the 95% confidence interval for the correlation)

Positive MUC1 localization at the cell membrane was present in many spindle-shaped and round cells in the annulus (Fig. 1a, b, and d). There were fewer positive cells noted in nucleus specimens (Fig. 1e). (Figure 1f presents a representative image from a negative control specimen.) In some instances, annulus cells showed a more general cytoplasmic localization pattern (Fig. 1c). In sites where there was a clear separation of the cells from the surrounding ECM, localization could be seen along the margin of the pericellular matrix (Fig. 1d). No localization was present in the territorial or interterritorial matrix of the annulus, or within the nucleus matrix. The percentage of cells positive for MUC1 localization was determined and statistically assessed for a relationship with disc grade; a positive significant correlation was present (*r* ^2^ = 0.473, *p* = 0.009), indicating that disc grade accounted for 47.3% of variation in the fraction of cells with MUC1 localization (Fig. 2).

**Fig. 3:**
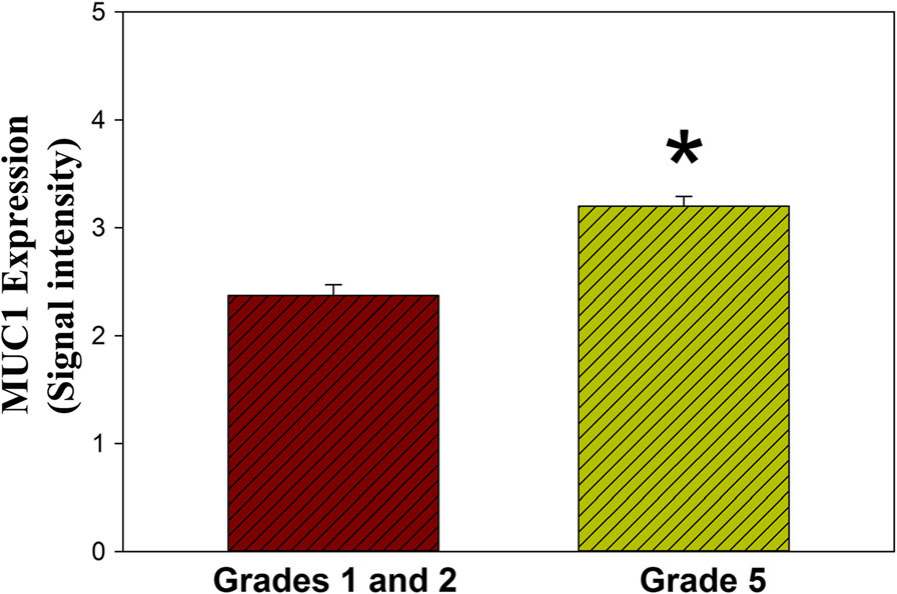
Graphical presentation of the significant increase in the relative MUC1 expression in vivo in more degenerated Thompson grade V disc tissue vs. healthier grade I and II discs (* *p* = 0.02; from Mann-Whitney evaluation)

Examination of molecular expression of MUC1 in human annulus tissue showed a small, but significant 1.2 fold upregulation in more degenerated, grade V discs compared to expression in healthier grade I and II discs (*p* = 0.02; Fig. 3; Table 1). No correlation was identified between the stages of disc degeneration and MUC1 levels in disc tissue.

**Fig. 4:**
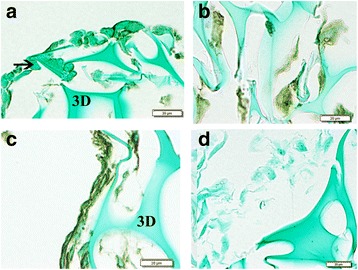
Representative images of cells cultured in 3D with immunolocalization of MUC1. **a**. Cells in the 3D construct under control; **b**. IL-1ß-treated cells; **c**. TNF-α-treated cells. “3D” marks the collagen sponge framework in which cells were cultured. **d.**  Representative image from a negative control. (*Arrows* mark cells which do not show localization. Bar = 10 μm)

**Fig. 5:**
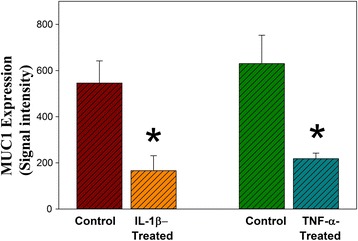
Graphical presentation of the relative MUC1 expression in control annulus cells compared to IL-1-ß or TNF-α treated cells cultured in 3D (* *p* = 0.0006 vs. relative control)

In vitro studies were carried to test the effect of exposure to proinflammatory cytokines on MUC1 expression. Human annulus cells were cultured in a 3D microenvironment for 14 days during which they were exposed to either control conditions or doses of IL-1beta (10^-2^ pM) or TNF-alpha (10^3^ pM). Dose levels had been optimized in previous studies [10, 12]. Immunohistochemistry identified MUC1 localization in many cells in the 3D construct under control (Fig. 4a), IL-1ß-treated cells (Fig. 4b) and in TNF-α-treated cells (Fig. 4c). (Figure 4d presents a representative negative control). Molecular analyses showed highly significant down regulation of MUC1 expression in cells exposed to IL-1ß or TNF-α (*p* = 0.0006; Fig. 5).

## Discussion

The present studies appear to be the first investigations of MUC1 in the human disc. MUC1 has previously been identified on the apical cell surface of epithelial cells, and is overexpressed in human carcinomas and hematologic malignancies in which it binds to caspase-8 and FADD, blocks caspase-8 recruitment, and thus prevents activation of the death receptor-induced extrinsic apoptotic pathway in cancer cells [2].

MUC1 is a surface-associated mucin with recognized signal transduction activity. Singh and Hollingsworth have recently reviewed the ways by which both the MUC1 extracellular and intracellular domains participate in cell signaling [20]. MUC1 has roles in several important signaling pathways, including Ras, ß-catenin, p120 catenin, p53, and estrogen receptor α [19, 23]. MUC1 may play an important part in the avascular disc by mechanisms modulating the effects of hypoxia signaling through HIF transcription factors. This MUC1 activity has been studied by Chaika et al. in pancreatic cancer cells, and the authors noted that MUC1 could be acting either by regulating HIF stability and it interaction partners, or by direct signaling through the MUC1 cytoplasmic tail using direct interaction with transcription factors [5, 20].

Data presented here showed an upregulation of MUC1 in more degenerated Thompson grade V compared to healthier grade I and II discs. In agreement with this was the positive correlation between the percentage of MUC1-positive cells and disc grade (*p* = 0.009); the high *r* ^2^ value for this correlation suggests that disc grade accounted for 47.3% of variation in the fraction of cells with MUC1 immunolocalization.

Our findings of the significant downregulation of MUC1 during IL-1ß and TNF-α exposure also suggest potential selective involvement of MUC1 during changes in the proinflammatory disc milieu during degeneration. As shown in the recent review by Risbud and Shapiro, we now know that there are numerous cytokines, in addition to IL-1ß and TNF-α, which undergo important changes during the process of disc degeneration ; these include IL-1α, IL-6 and IL-17 (see [18] for a recent review). Our novel findings here with MUC1 and its increasing expression during disc degeneration point to the importance of future work which focuses upon interactions of MUC1 with major cytokines and chemokines in the degenerating disc.

Oxidative stress-induced apoptosis is also an important event in the aging and degenerating disc which puts an already small cell population at further risk for a decrease in cell numbers [11]. Both in vivo and in vitro research has focused on the modulation of apoptosis by MUC1 expression [20]. Studies have shown that MUC1 positive cells were more sensitive to apoptosis induced by FasL compared to MUC1 negative cells; the proposed mechanism was upregulation of the cell surface Fas receptor by increased intracellular trafficking [6].

As previously noted, MUC1 signaling in tumors has been found to reprogram transcription of connective tissue growth factor CTGF, which is a strong mediator of extracellular matrix (ECM) remodeling and angiogenesis [3]. In the present work, we identified a significant upregulation in MUC1 expression in annulus tissue from more degenerated grade V discs compared to healthier grade I-II discs (*p* = 0.02). Abbot et al. have previously shown that the stage of degeneration of the nucleus pulposus can influence the nucleus cell response to CTGF [1], suggesting another potentially important role for MUC1 in annulus cells.

Unfortunately, we were unable to carry out qRT-PCR molecular studies on cells/tissue described here because of insufficient amounts of remaining mRNA. Future research on the role of MUC1 in disc aging and degeneration appears warranted to determine whether MUC1 might act as a regulator in the hypoxic environment, helping disc cells to survive under hypoxic conditions by stabilization and activation of HIF-1α (as shown in pancreatic cancer cells by Chaika et al. [5], or by exerting an influence on the response to CTGF. We look forward to future studies which should include qRT-PCR gene expression analysis, and an expanded exploration of the relationship of MUC1 to disc aging and degeneration.

## Conclusion

This is the first study describing the constitutive presence of MUC1 in the human disc, and its increased expression during disc degeneration, and its in vitro downregulation during exposure to the proinflammatory cytokines IL-1ß and TNF-α. Since MUC1 may have an important role in disc aging and degeneration by acting as a regulator in the hypoxic environment (helping disc cells to survive under hypoxic conditions by stabilization and by activation of HIF-1α as previously recognized in pancreatic cancer cells), we look forward to future studies on the role of MUC1 in disc aging and degeneration.

## Abbreviations

CHTN: Cooperative Human Tissue Network
CTGF: Connective tissue growth factor, also called CCN2
HIF-1: Hypoxia inducible factor-1
IL-1ß: Interleukin-1 beta
MUC1: Mucin 1
TNF-α: Tumor necrosis factor-alpha
